# Pharmacokinetic and pharmacodynamic modeling of sarafloxacin against avian pathogenic *Escherichia coli* in Muscovy ducks

**DOI:** 10.1186/s12917-017-0964-0

**Published:** 2017-02-10

**Authors:** Yang Yu, Yu Feng Zhou, Jian Sun, Wei Shi, Xiao Ping Liao, Ya Hong Liu

**Affiliations:** 1 National Risk Assessment Laboratory for Antimicrobial Resistance of Animal Original Bacteria, South China Agricultural University, Guangzhou, 510642 China; 2 Guangdong Provincial Key Laboratory of Veterinary Pharmaceutics Development and Safety Evaluation, South China Agricultural University, Guangzhou, 510642 China; 3 Laboratory of Veterinary Pharmacology, College of Veterinary Medicine, South China Agricultural University, Guangzhou, Guangdong 510642 China; 4 College of Veterinary Medicine, National Reference Laboratory of Veterinary Drug Residues, South China Agricultural University, Guangzhou, 510642 China

**Keywords:** Dosage regimens, Muscovy ducks, Mutant prevention concentration, Pharmacokinetics/pharmacodynamics, Sarafloxacin, Avian pathogenic *Escherichia Coli*

## Abstract

**Background:**

This study focused on utilizing pharmacokinetics/pharmacodynamics (PK/PD) modeling to optimize therapeutic dosage regimens of sarafloxacin against avian pathogenic *Escherichia. coli* O78 strain in Muscovy ducks. The ex vivo PK/PD study of sarafloxacin was conducted in Muscovy ducks after intravenous (i.v.) and oral (p.o.) administrations at a single dose of 10 mg/kg bodyweight (BW). The serum samples were analyzed by reverse phase high-performance liquid chromatography (RP-HPLC) using a fluorescence detection method. Sarafloxacin PK data were analyzed by a non-compartmental method using Winnonlin software.

**Results:**

Calculations of the area under the concentration-time curves (AUC_0-24h_) were 8.57 ± 0.59 and 8.37 ± 0.29 μg · h/ml following i.v. and p.o. administration, respectively. Elimination half-lives (*t*_*1/2*β_) were 6.11 ± 0.99 h and 8.21 ± 0.64 h for i.v. injection and p.o. administration, respectively. The mean in vitro plasma protein binding of sarafloxacin was 39.3%. Integration using the sigmoid *E*_max_ model, the mean values of AUC_0-24h_/MIC needed for bacteriostatic, bactericidal and bacterial eradication action were 25.4, 40.6, and 94.4 h, respectively.

**Conclusions:**

Sarafloxacin administered at a 10 mg/kg dose may be insufficient for treatment of *E. coli* O78 infections with an MIC equally to or over 0.125 μg/ml. Furthermore, higher doses of sarafloxacin are required to minimize antimicrobial resistance considering the MPC theory.

## Background

The minimum inhibitory concentration (MIC) and minimum bactericidal concentration (MBC) were used to evaluate the efficacy of antimicrobial drugs to eradicate the entire pathogen population [1]. The mutant prevention concentration (MPC) has been proposed to evaluate the ability of antimicrobial agents to prevent the emergence of antibacterial resistance [2]. Pharmacokinetics/pharmacodynamics (PK/PD) principles may provide an effective way to control the occurrence and dissemination of bacterial resistance [3, 4]. For fluoroquinolones, the PK/PD indices are calculated by the ratio of area under the concentration-time curve to MIC (AUC/MIC) and the ratio of peak drug concentration to MIC (C_max_/MIC) [3, 5]. Recently, the values of AUC/MPC and C_max_/MPC were determined to minimize the occurrence of the antibacterial resistance [1, 6, 7].

Muscovy ducks are reared for their distinctive taste, high yield of breast meat and low calorie content [8]. Colibacillosis is the most common disease in the poultry industry causing serious death and economic losses worldwide, e.g. in China [9]. Birds infected with Avian pathogenic *Escherichia coli* (APEC) are more likely to develop infectious bursal disease, mycoplasmosis, coccidiosis, Newcastle Disease, infectious bronchitis, and nutritional deficiencies [10]. APEC are responsible for aerosacculitis, polyserositis, pericarditis, yolk sac infection, respiratory tract infection, septicemia and other extra-intestinal diseases in chickens, turkeys, ducks and other avian species [11–13]. Serotype O78 is recognized as one of the most common and prevalent serogrouping of APEC isolates [11, 12]. *E. Coli* strains with the O78 serotype are highly pathogenic and commonly exist in poultry industry. Therefore, treatment of colibacillosis caused by APEC O78 demands far more attention in poultry farming. However, current vaccines have poor efficacy, and therapy is mainly based on antimicrobial therapy.

Sarafloxacin, a second generation fluoroquinolone antibiotic exclusively developed for veterinary use, shows excellent antimicrobial activity [14–16], and is mainly used to treat colibacillosis by binding to topoisomerase II in Gram-negative bacteria [17–19]. In the European Union, sarafloxacin was proposed for use in poultry and fish in 1998 [20]; and difloxacin (whose primary metabolite is sarafloxacin) was authorized in 1998 but in the meantime the marketing authorization was withdrawn [21].

Publications have reported the pharmacokinetics of sarafloxacin in several animals and birds [15, 22]; however, few studies focused on the PK/PD modeling of sarafloxacin in Muscovy ducks. The aims of this work are to: 1) study the PK/PD modeling of sarafloxacin; 2) optimize the clinical dosage regimens for treatment of colibacillosis in Muscovy ducks particularly against the *E.coli* O78 strains; and 3) assess the risks of using sarafloxacin employing the MPC theory.

## Methods

### Bacterium and reagents

The experiments were performed using an APEC O78 strain (identified by a serum agglutination test) isolated from ducks colibacillosis and stored in our laboratory. Phylogenetic grouping of this strain was B_2_ type and determined by multiplex PCR using specific primers from *chuA*, *yjaA* genes and TSPE4.C2 fragment. *E. coli* ATCC 25922 (China Institute of Veterinary Drug Control, Beijing, China) was used for the quality control purpose. Sarafloxacin was purchased from Unichna Bio-technology of Zhengzhou Co., LTD, Zhengzhou, China.

### Animals and experimental design

Twenty-four healthy Muscovy ducks (purchased from the Jiangcun poultry market in Baiyun district, Guangzhou, China) of 100–120 days old and weighing 2.4–2.6 kg were used. Ducks were fed commercial drug-free food. All ducks were examined daily, adapted well to the environment, and were clinically healthy prior to the study. All protocols were approved by the Animal Research Committees of South China Agricultural University. All experiments were conducted using the guidelines of the American Association for Accreditation of Laboratory Animal Care (AAALAC) [23].

Animals were randomly divided into two groups of 12/group. Feed was eliminated for 12 h before and 6 h post-administration. Sarafloxacin (2.5%) was injected intravenously into the left brachial vein at 10 mg/kg body weight (BW). For oral administration, a soluble powder (5%), was directly administered into the crop at 10 mg/kg BW. Blood samples were collected from the right brachial vein at 0, 5, 15, 30, 45 min, and 1, 1.5, 2, 3, 4, 6, 8, 12, 16, 24, 36 h post-i.v. administration. For p.o. administration, the time points for blood collection were at 0, 10, 20, 30, 45 min, and 1, 1.5, 2, 3, 4, 6, 8, 12, 16, 24, 36 h. The serum samples were obtained by centrifugation at 2000 g for 10 min and stored at − 20 °C until analyses within 2 weeks.

### Sarafloxacin analysis

A total of 0.1 mL acetonitrile was added into 0.1 mL of serum sample. The mixture was rigorously vortexed for 1 min, and then centrifuged at 15,000 g for 5 min. The supernatant was filtered through a 0.22 μm cellulose membrane, and 10 μL were injected into the reverse phase high-performance liquid chromatography (RP-HPLC) system. This RP-HPLC includes a 2695 Waters Alliance system (Milford, MA, USA), a HYPERSIL BDS C_18_ Column (5, USA), and a HYPERS. The mobile phase consisted of acetonitrile and 0.1% trifluoroacetic acid (29:71, v: v) at 1 mL/min flow rate. A Waters 2475 fluorescence detector operated at an excitation wavelength of 280 nm and an emission wavelength of 460 nm.

The retention time of sarafloxacin in the serum was 4.9 ± 0.2 min. The standard curve of sarafloxacin was linear in the range of 0.02–10 μg/mL with correlation coefficient (R) of 0.994. The recovery of sarafloxacin from serum ranged from 96.5 ± 1.45% to 104 ± 0.76%. The limit of detection (LOD) and limit of quantitation (LOQ) were 0.01 and 0.02 μg/mL, respectively.

### Protein binding

In vitro protein binding was determined using an ultracentrifugation method [24]. Sarafloxacin was added to blank plasma samples to produce concentrations of 0.1, 1 and 10 μg/mL. After 1 h incubation at 37 °C, 0.5 mL of each sample was placed in a Microcon YM-10 centrifugal filter device (Millipore, USA), and centrifuged at 1500 g for 20 min at 4 °C. Nonspecific binding of the drug to the ultrafiltration membrane was also determined. The drug concentrations in drug-free plasma samples and ultrafiltrate samples were determined as described above. The in vitro plasma protein binding of sarafloxacin was calculated using the following equation:

$$ \mathrm{B}\mathrm{P}\%=\left[1-{\mathrm{C}}_{\mathrm{U}}/{\mathrm{C}}_{\mathrm{T}}\right]\times 100\% $$

where C_U_ is the concentration in the ultrafiltrate, and C_T_ is the total drug concentration.

### MIC, MBC and MPC

The MIC and MBC were determined in both Mueller Hinton Broth (MHB) and drug-free serum using a standard broth micro-dilution method [25]. MPC was determined as previously described with slight modifications [26]. A single colony was grown overnight in 10 mL MHB, and 1 mL of this culture was then transferred to 100 mL MHB for 24 h incubation. The culture was washed twice with PBS and centrifuged at 4000 g for 20 min at 4 °C. The precipitate was re-suspended in 10 mL of MHB to obtain a 10^10^ CFU/mL bacterial load. Aliquots of 100 μL were re-suspended and applied to MHA plates containing sarafloxacin at concentrations of 1× to 128× MIC. MPC was recorded as the lowest concentration that prevented bacterial growth after 72 h incubation.

### Time-killing curves

In vitro time-killing curve experiments were conducted in MHB and blank serum using drug concentrations ranged from 0.25 × to 16 × MIC. Serum samples obtained at 0, 0.5, 1, 2, 4, 6, 8, 12 and 24 h post-p.o. administration were used to measure the ex vivo time-killing curves. The initial bacterial counts were about 10^6^ CFU/mL. The 50 μL aliquot was diluted appropriately, and 100 μL of each dilution were applied onto MHA plates at 0, 3, 6, 9 and 24 h. All agar plates were incubated at 37 °C for 18 h. The limit of detection (LOD) was 100 CFU/mL.

### PK analysis

Pharmacokinetic data were analyzed using the Winnonlin Program (version 5.1; Pharsight, St. Louis, MO, USA) based on a non-compartmental model. The bioavailability was estimated by the following equation:

$$ \mathrm{F}\%={\mathrm{AUC}}_{\mathrm{PO}}/{\mathrm{AUC}}_{\mathrm{IV}}\times 100\% $$

where AUC_PO_ is the AUC after p.o. administration, and AUC_IV_ is the AUC after i.v. injection.

### PK/PD integration and modeling

The PK/PD modeling was analyzed by the Sigmoid E_max_ model of Winnonlin Software. The equation was defined as:

$$ E={E}_{max}-\left({E}_{max}-{E}_0\right)\times {C}^N/\left({C}^N+ E{C_{50}}^N\right) $$

Where *E* is the antibacterial effect. *E*_max_ is the log_10_ difference in bacterial counts between 0 and 24 h in the control sample. *E*_0_ is the log_10_ difference in bacterial counts between 0 and 24 h in the sample containing sarafloxacin when the LOD of 100 CFU/mL is reached. *C* is the PK/PD indexes. EC_50_ is the PK/PD surrogate parameters producing 50% of the maximal antibacterial. *N* is the Hill coefficient which describes the slope of the sigmoid curves.

The PK/PD surrogate markers of antibacterial activity, including C_max_/MIC, AUC_0-24h_/MIC, C_max_/MPC and AUC_0-24h_/MPC, were determined using in vitro PD data and in vivo PK values post-i.v. and -p.o. administration of sarafloxacin. In this model, AUC_0-24h_/MIC values were calculated according to the four levels of antibacterial effectiveness: a bacteriostatic action (i.e., no change in colony counts, *E* = 0), a 50% reduction in colony counts, a bactericidal action (i.e., a 99.9% reduction in bacterial counts, *E* = −3), and a bacterial elimination effect (i.e., a 99.99% reduction in bacterial counts, *E* = −4) [27].

### Evaluation of dosage regimens

Optimal dosage providing a specific antibacterial effect was calculated using the following equation:

$$ \mathrm{Dose}\ \left(\mathrm{per}\ \mathrm{day}\right) = {\mathrm{Cl}}_{\left(\mathrm{per}\ \mathrm{hour}\right)}\times \left({\mathrm{AUC}}_{0\hbox{-} 24\mathrm{h}}/\mathrm{MIC}\right)\times \mathrm{MIC}/\left( F\times fu\right) $$

Where Cl is the clearance, AUC_0-24h_/MIC is the ratio of the area under serum concentration-time curve over a 24 h period to MIC value achieved from PK/PD integration, *F* is the bioavailability, *fu* is the unbound fraction of sarafloxacin.

### Statistical analysis

The PK and ex vivo PK/PD data are presented as mean ± SD. All the data are conducted using the SPSS 16.0 software package (SPSS Inc., Chicago, IL).

## Results

### Pharmacokinetic profiles

**Fig. 1:**
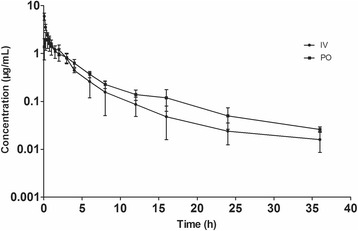
Semi-logarithmic plot of serum concentrations *v.s.* time of sarafloxacin in Muscovy ducks (*n* = 12) following a single i.v. and p.o. administration at a single dose of 10 mg/kg BW. Bars represent standard deviations

A Semi-logarithmic plot of serum concentrations of sarafloxacin following i.v. and p.o. administrations is presented in Fig. 1 and PK parameters calculated by Winnonlin using a non-compartmental model are shown in Table 1. Initial drug concentration in serum for i.v. injection was about 7.53 μg/mL, and it rapidly decreased to approximately 0.2 μg/mL at 12 h. For oral administration, a rapid absorption, with a time of peak concentration (*T*_*max*_) of 0.44 ± 0.16 h, was observed and the *C*_*max*_ was 2.03 ± 0.73 μg/mL. The half-lives of elimination (*t*_*1/2*β_) were 6.11 ± 1.97 and 8.21 ± 2.31 h following i.v. and p.o. administrations, respectively and the AUC_0-24h_ were 8.43 ± 1.06 and 8.26 ± 1.08 μg · h/mL, respectively. Bioavailability (F%) for oral dosing route was 97.6 ± 5.32%. Drug concentrations of both routes of administration were below the LOD level at 48 h after a single dose of sarafloxacin in Muscovy ducks.

**Table 1:** Pharmacokinetic parameters of sarafloxacin after a single i.v. and p.o. administration at a dose of 10 mg/kg BW to Muscovy ducks (*n* = 12) using a non-compartmental model

Parameter	Unit	IV	PO
T_1/2β_	h	6.11 ± 1.97	8.21 ± 2.31
T_max_	h	—	0.44 ± 0.16
C_max_	μg/mL	—	2.03 ± 0.73
AUC_0-24 h_	μg/mL · h	8.43 ± 1.06	8.26 ± 1.08
V_Z_	L/kg	10.4 ± 3.32	—
Cl	L/kg · h	1.18 ± 0.16	—
V_Z_/F	L/kg	—	14.1 ± 3.55
Cl/F	L/kg · h	—	1.21 ± 0.17
MRT_0-24 h_	h	4.17 ± 1.04	7.75 ± 1.86
F	%	—	97.6 ± 5.32

*T*_*1/2*β_ elimination half-life, *AUC* area under the curve, *T*_*max*_ time for peak concentration, *C*_*max*_ peak concentration, *V*_*Z*_ */F* apparent volume of distribution, *Cl/F* overall body clearance, *MRT* mean residence time, *AUMC* area under the moment curve, *F* bioavailability

### Protein binding

The percent of sarafloxacin recovery from ultrafiltration membrane exceeded 95%, suggesting that the ultrafiltration membrane had no specific adsorption of the drug. Table 2 shows the protein binding rates of sarafloxacin. Bound fraction of sarafloxacin ranged from 24.5 to 56.1% for drug concentrations varied from 0.1 to 10 μg/mL with a mean binding rate of 39.3%.

**Table 2:** Plasma protein binding of sarafloxacin at three spiked levels

Spiked levels (μg/mL)	Protein binding rate (%)
0.1	56.1 ± 4.58
1	37.4 ± 2.07
10	24.5 ± 0.90

### MIC, MBC and MPC values

The MIC, MBC and MPC of sarafloxacin are presented in Table 3. The MPC value for sarafloxacin was 1 μg/mL. The ratio of MBC/MIC was 2 μg/mL either in MH broth or in serum, and MPC/MIC value was 8 in MH broth medium.

**Table 3:** MIC, MBC and MPC values of sarafloxacin against *E. coli* O78 in MHB and serum medium

Parameter	Values (μg/mL)	Parameter	Values (μg/mL)
*In MH broth*		*In serum*	
MIC	0.125	MIC	0.25
MBC	0.25	MBC	0.5
MPC	1	MBC/MIC	2
MBC/MIC	2		
MPC/MIC	8		

*MIC* minimum inhibitory concentration, *MBC* minimum bactericidal concentration, *MPC* mutant prevention concentration, *MBC/MIC* ratio of in vitro MBC to MIC, *MPC/MIC* ratio of in vitro MPC to MIC

### Time-killing curves

**Fig. 2:**
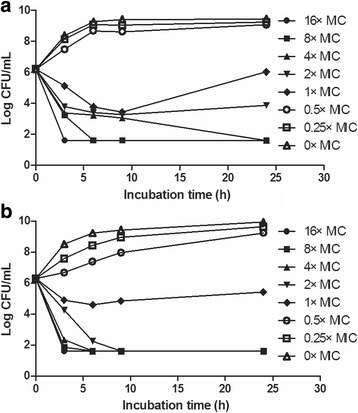
In vitro time-kill curves of sarafloxacin against the *E. coli* O78 strain in serum (**a**) and MHB (**b**). Numerical values on the right represent sarafloxacin concentrations

In vitro time-killing curves of sarafloxacin against are shown in Fig. 2. Bacterial growth was inhibited obviously at drug concentration at 1 × MIC, but re-growth was observed after 9 h of incubation. However, the number of bacteria after incubation for 24 h was still below the initial load. For exposure at drug concentration at 2 × MIC, a bactericidal activity was observed in 24 h, but bacterial cells were not eradicated in the serum samples. When drug concentration was raised from 4 × to 16 × MIC, better antibacterial effectiveness was achieved, showing both bactericidal and eradication effect in 3 h of incubation.

**Fig. 3:**
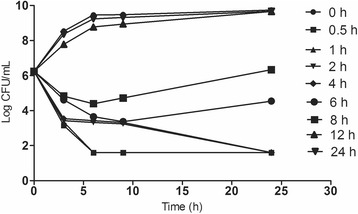
Ex vivo time-kill curves of sarafloxacin against the *E. coli* O78 strain after p.o. administration of sarafloxacin at a dose of 10 mg/kg BW. Values are the means (*n* = 12) and the SD values are excluded for clarity

Figure 3 shows the ex vivo time-killing curves, in which sarafloxacin concentrations in serum samples obtained at 0, 0.5, 1, 2, 4, 6, 8, 12 and 24 h after p.o. administration were 0, 2.22, 1.68, 1.32, 0.75, 0.37, 0.27, 0.13, and 0.04 μg/mL, respectively. A rapid bactericidal or elimination action was observed after 3 h of incubation and no re-growth was seen after 24 h for all serum samples collected between 0.5 and 6 h. A bacteriostatic action could be observed from serum samples collected at 8 h, whereas the serum sampling at 12 h and 24 h exhibited almost non-inhibitory effect.

### PK/PD integration and modeling

**Fig. 4:**
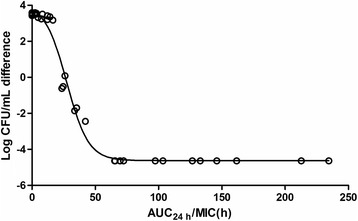
Plots of the ex vivo AUC_0-24h_/MIC ratios versus the difference of bacterial counts (log _10_ CFU/mL) between 0 and 24 h for *E. coli* O78 strain

PK/PD parameters from the study are summarized in Table 4. The values of AUC_0-24h_/MIC and C_max_/MIC were 33.4 ± 4.44 h and 8.12 ± 2.92. Table 5 shows the indexes of PK/PD integration from Sigmoid E_max_ model. The relationship between antibacterial effectiveness and PK/PD parameter of AUC_0-24h_/MIC is exhibited in Fig. 4. The mean values of AUC_0-24h_/MIC that produced bacteriostatic action, bactericidal activity and bacterial eradication effect were 25.4, 40.6 and 94.5 h, respectively.

**Table 4:** Integration of PK/PD parameters of sarafloxacin against *E. coli* O78 after i.v. and p.o. administration (10 mg/kg) in ducks

Parameter	IV	PO
AUC_0-24 h_/MIC (h)	34.3 ± 4.72	33.5 ± 4.44
C_max_/MIC	-	8.12 ± 2.92
AUC_0-24 h_/MBC (h)	17.2 ± 2.36	16.7 ± 2.23
C_max_/MBC	-	4.06 ± 1.45
AUC_0-24 h_/MPC (h)	8.57 ± 1.18	8.37 ± 1.11
C_max_/MPC	-	2.03 ± 0.73

**Table 5:** Parameters of PK/PD modeling based on ex vivo serum data after p.o. administration of sarafloxacin in muscovy ducks (*n* = 12)

Parameter	Unit	*E. coli* O78
Log *E*_0_	CFU/mL	3.58 ± 0.27
Log *E*_0_ − Log *E*_max_	CFU/mL	8.31 ± 0.28
Slope(*N*)	—	3.42 ± 0.15
AUC_0-24 h_/MIC for bacteriostatic action	h	25.4 ± 1.68
AUC_0-24 h_/MIC EC_50_	h	27.5 ± 1.86
AUC_0-24 h_/MIC for bactericidal action	h	40.6 ± 2.46
AUC_0-24 h_/MIC for bacterial eradication	h	94.5 ± 7.70

*E*_0_: difference in bacterial count in control sample between time 0 and 24 h; *E*_max_: difference in bacterial count in sample containing sarafloxacin between time 0 and 24 h, when limit of detection is reached; *N*: the Hill coefficient; AUC_0-24 h_/MIC EC_50_: AUC_0-24 h_/MIC of drug producing 50% of the maximum antibacterial effect

## Discussion

Sarafloxacin MICs in MHB or serum against the *E. coli* O78 strain used in this study were 0.125 ~ 0.25 μg/mL, which is moderate susceptible comparing with previous reports [14, 28]. In this study, a low ratio of MBC/MIC suggests that sarafloxacin has bactericidal activity against the *E. coli* O78 strain. In addition, the MPC/MIC ratio for sarafloxacin was in agreement with the values calculated for fluoroquinolones from previous reports [29].

In Muscovy ducks, sarafloxacin was rapidly and extensively distributed into body fluids and tissues after i.v. administration. A calculated distribution volume was 10.04 ± 3.32 L/kg, which is higher than that in broilers [15]. The *t*_*1/2*β_ after i.v. injection of sarafloxacin was estimated to be 6.11 ± 1.97 h, much higher than those reported in pigs and chickens [15], and a slightly higher than that of marbofloxacin in ducks [30]. Therefore, a longer withdrawal time may be required in Muscovy ducks than in other food-producing animals for consideration of residue food safety concerns. A maximum concentration (*C*_*max*_) of 2.03 ± 0.73 μg/mL following p.o. administration of sarafloxacin in this study was observed at 0.44 ± 0.16 h. Bioavailability was 97.6% in ducks, which was much higher than the previously reported values of 42.6% in pigs, 59.6% in chickens [15], but similar to that of marbofloxacin and moxifloxacin in ducks [30, 31], indicating a rapid and almost complete absorption following p.o. administration. Total body clearance was 1.18 L/kg/h, similarly to that in broilers [15]. The AUCs after i.v. and p.o. administrations were comparable with the results obtained from chickens and pigs [15].

It is recognized that only the unbound fraction of the drug has the antibacterial activity [27]. The plasma protein binding of sarafloxacin was 39.3%, consistent with previous reports [24]. Based on the time-killing curves, antibacterial activity of sarafloxacin against the *E. coli* strains was concentration-dependent. Similar to other fluoroquinolones and aminoglycosides, the ratios of AUC_0-24h_/MIC and C_max_/MIC were key PK-PD parameters correlating with clinical efficacy in target animals [3]. Attainment target of AUC_0-24h_/MIC of 100–125 h or C_max_/MIC of 8–10 should be obtained for fluoroquinolones in order to exert great bacteriological and treatment effects against Gram-negative bacterial pathogens; on the contrary, AUC_0-24h_/MIC of 30 h should be achieved against Gram-positive pathogens [5, 32, 33]. However, optimal PK/PD endpoints for fluoroquinolones were not fixed dependent upon combinations of drug, pathogen and target species, as described in previous reports [16, 28]. Thus, appropriate endpoints of PK/PD parameters should be established for specific drug against specific pathogen in given target animal species. In the present study, the values of AUC_0-24h_/MIC theoretically needed for bacteriostatic, bactericidal and eradication activity were calculated using the sigmoid *E*_max_ model. The value calculated for eradication activity against the *E. coli O78* strain (94.5 h) was smaller than the literature value of 125 h [32]. At a dosage of 10 mg/kg BW administered p.o., the in vivo AUC_0-24h_/MIC ratio for the *E. coli* O78 strain (33.5 h) was slightly lower than the ex vivo AUC_0-24h_/MIC ratio required for bactericidal action (40.6 h). These results indicated that the dosage of 10 mg/kg BW was not high enough to cure colibacillosis caused by *E. coli* O78 (MIC = 0.125 μg/mL). Thus, daily dosage regimens for sarafloxacin following p.o. administration to achieve bacteriostatic, bactericidal and eradicaton effects were calculated as 6.30, 10.1 and 23.6 mg/kg BW, respectively for dose optimization*.* Considering that sarafloxacin is concentration dependent antibiotic and convenience of clinical application, single dose administration daily is recommended.

As described in other previous reports [1, 6, 7], the MPC theory is also taken into consideration when optimizing the sarafloxacin dosage regimens in ducks. Traditional PK/PD modeling regards MIC and MBC as the major PD parameters; however, they may have only considered antibacterial activity against pathogenic population [1]. With the increasing of antimicrobial resistance, the MPC and mutant prevention window (MSW, defined as the concentration range between MIC and MPC in which the resistant mutants are selectively enriched [2]) have been used to evaluate antibacterial activity to prevent selection and development of antibacterial resistant mutants. The new PK/PD parameters, including AUC_0-24h_/MPC, C_max_/MPC and T > MPC, were used for the optimization of dosage regimens in the current study. In this study the values of AUC_0-24h_/MPC and C_max_/MPC (8.37 h and 2.03, respectively) for sarafloxacin against the *E. coli* O78 strain were comparable to the values (9.76 h and 1.26) obtained for enrofloxacin against *Pasteurella multocida* in buffalo calves [1] and the values (9.70 h and 1.40) obtained for marbofloxacin against *E. coli* [34]. According to previous reports, the values of AUC_0-24h_/MPC of 9 to 12 h could prevent occurrence of resistant mutants for marbofloxacin against *E. coli* [35], which means average plasma concentration being 37.5 ~ 50% of MPC value can achieve a efficacy of preventing resistant mutants based on the dimensional perspective of AUC/MIC theory [36]. In order to cure diseased animals and control selection and development of drug resistance, the drug concentration in serum should exceed the MPC value as long as possible [7]. In this study, the value of T > MPC for sarafloxacin was 1.85 h, which was short due to high MPC. Similarly, AUC_0-24h_/MPC and C_max_/MPC from this study were lower than the values reported in literature for preventing the selection of resistant mutants. Therefore, the AUC/MPC of 8.37 h of sarafloxacin may be insufficient for preventing resistant mutant in the current work. Further study is needed to better understand the PK/PD characteristic for sarafloxacin, considering the interaction of drugs, pathogens and animal species.

## Conclusions

To our knowledge, it is the first report about the PK/PD modeling study for sarafloxacin in Muscovy ducks targeting avian pathogenic *E. coli* O78 strain. Ex vivo PK/PD modeling and the MPC theory were used to optimize reasonable dosage regimens. Our findings suggested that a single dose of 10 mg/kg BW administered orally is insufficient to treat infection caused by *E. coli* O78 with an MIC of 0.125 or higher. In addition, a higher dose of sarafloxacin is needed to minimize occurrence of antimicrobial resistance with consideration of MPC theory.

## Abbreviations

i.v.: Intravenous
LOD: Limit of detection
LOQ: Limit of quantification
MBC: Minimal bactericidal concentration
MIC: Minimal inhibitory concentration
MPC: Mutant prevention concentration
p.o.: Per os
PK/PD: Pharmacokinetics and pharmacodynemics
